# pyWitness 1.0: A python eyewitness identification analysis toolkit

**DOI:** 10.3758/s13428-023-02108-2

**Published:** 2023-07-19

**Authors:** Laura Mickes, Travis M. Seale-Carlisle, Xueqing Chen, Stewart Boogert

**Affiliations:** 1 School of Psychological Science, University of Bristol, Bristol, UK; 2 School of Psychology, King’s College, University of Aberdeen, Aberdeen, UK; 3 Department of Physics and Astronomy, University of Manchester, Manchester, UK

**Keywords:** Eyewitness, Memory, Receiver operating characteristic, Confidence accuracy characteristic, Signal detection theory, Detection-plus-localization, Recognition memory, Visual search task

## Abstract

pyWitness is a python toolkit for recognition memory experiments, with a focus on eyewitness identification (ID) data analysis and model fitting. The current practice is for researchers to use different statistical packages to analyze a single dataset. pyWitness streamlines the process. In addition to conducting key data analyses (e.g., receiver operating characteristic analysis, confidence accuracy characteristic analysis), statistical comparisons, signal-detection-based model fits, simulated data generation, and power analyses are also possible. We describe the package implementation and provide detailed instructions and tutorials with datasets so that users can follow. There is also an online manual that is regularly updated. We developed pyWitness to be user-friendly, reduce human interaction with pre-processing and processing of data and model fits, and produce publication-ready plots. All pyWitness features align with open science practices, such that the algorithms, fits, and methods are reproducible and documented. While pyWitness is a python toolkit, it can also be used from R for users more accustomed to this environment.

## Introduction

Recognition memory is used by eyewitnesses to crimes when identifying perpetrators from identification (ID) procedures. ID procedures always include the police suspect. The suspect may be the perpetrator of the crime in question or an innocent person. ID procedures vary in the number of people: from 1, in the case of a procedure called a ‘showup,’ to 2 or more (6 is common in the US), in the case of a ‘lineup.’ The other people in lineups are ‘fillers’ and are known not to be involved in the crime. Fillers are used to populate the lineup because they physically resemble the description of the perpetrator or the suspect (e.g., Wells et al., 2020). In the real world, showups are typically administered where the suspect is viewed in person and lineups are administered using photos or videos of the suspect and fillers. In the lab, researchers most often test memory by using photos, even for showups (e.g., Gronlund et al., 2012). And, unlike the police, researchers know whether the suspect is innocent or guilty.

Eyewitnesses attempt to identify the person they saw involved in the crime from a showup or lineup. In the lab, participant eyewitnesses typically watch a video of a mock crime and then try to identify the actor perpetrating the crime in the video. With showups, they either affirm that the suspect is the person who committed the crime or not. The potential correct outcomes are correct IDs and correct rejections. A correct ID occurs with the ID of the guilty suspect and a correct rejection occurs when the eyewitness does not identify the innocent suspect. The possible incorrect outcomes are false IDs and misses. A false ID occurs by identifying the innocent suspect and a miss occurs by not identifying the guilty suspect. With lineups, the same decisions and outcomes are possible, and because there are also fillers, eyewitnesses can make filler IDs. Filler IDs, of course, are always incorrect.

Over the last decade, receiver operating characteristic (ROC) analysis has become a standard analysis because it measures discriminability (the ability to distinguish innocent from guilty suspect) separate from response bias (the likelihood of identifying or not identifying a lineup member) (Wixted and Mickes , 2012; Albright and Rakoff , 2020). Confidence-based ROC analysis is a graphical analysis that plots correct ID and false ID rates for every level of confidence. Another graphical analysis that is now commonly conducted on lineup and showup data is confidence accuracy characteristic (CAC) analysis (Mickes , 2015). CAC analysis measures the likelihood the identified suspect is the perpetrator at each level of confidence. This analysis plots suspect ID accuracy as a function of confidence.

To understand eyewitnesses’ performance on identification procedures, researchers consider results from several different types of analyses (i.e., ROC and CAC analyses). They often use eclectic software, including MATLAB, R, Excel, in piece-meal fashion. This common but less-than-ideal approach can have multiple undesirable consequences. Such downsides include being prone to errors made at any stage of analyses that then get propagated through the analysis pipeline. Another downside is that way of working makes data sharing and analysis replication difficult. Thus, communication between researchers is limited. pyWitness solves these problems by harmonizing and streamlining the analyses of data from experiments involving lineups, showups, and list-learning (standard recognition memory experiments). There is an R package called “signal detection theory lineup” (sdtlu; Cohen et al., 2021). With sdtlu, researchers can perform certain analyses and model fits on lineup data. There is some overlapping functionality between sdtlu and pyWitness, but the main differences are the available models that each package supports, the possibility of extension, and usability. Recently an R shiny app, powe(R)OC, was made available to perform power analyses for eyewitness ROCs (Mah , 2022). These technological solutions will advance the way researchers conduct eyewitness ID analyses.

We developed pyWitness to be user-friendly with a flexible internal data format that requires as little human interaction as possible (e.g., manipulating data, transcribing values, etc.). Importantly, it stores all of the relevant algorithms, fits, and methods in a reproducible and documented way, promoting open science practices. Throughout this paper, we denote the code in a fixed-width font (e.g., example code). To bolster usage, we provide an online manual, an issue tracker, and a forum for users (available at https://lmickes.github.io/pyWitness/). Users with any level of programming experience can analyse and fit complex models to data and produce publication-ready figures and tables.

pyWitness is written in python, an up-to-date object-oriented programming language. Python is simple to extend to include new input data formats and add new models. The code replicability is ensured by using unit tests and git. Another benefit of python is an educational one because users can obtain experience with a widely used programming language. Users unfamiliar with python but familiar with R or MATLAB, for example, should be able to easily use and extend pyWitness. For users who still prefer using R, they can use it directly in R via the reticulate library.

Python classes and objects allow a high level interface to processing, enabling users to perform analyses with a single line of code which might have taken hours in Excel, for example. Therefore, pyWitness can free time that can be spent on the scientific questions rather than on data manipulation and analyses. pyWitness’s high-level interfaces allow researchers to innovate and quickly create new analyses, particularly those that involve signal detection-based models. A well-curated analysis framework, like pyWitness, has the potential to create a community of users that work collectively on analyses and modelling.

### Code overview and structure

pyWitness follows a object-oriented design, where data and functions are gathered together in high-level classes. pyWitness is a Python module consisting of three base classes. The classes are DataRaw, DataProcessed, and ModelFit. Each class represents a stage of data processing and contains one or more internal pandas.DataFrame to store the data. Each class also has functions that operate on the internal data to perform useful analysis operations. By keeping the underlying data as a pandas.DataFrame allows users to perform complex data analysis steps just using pandas. The inclusion of these interfaces allows for flexibility in adding features to future versions of pyWitness. All data critical for standard analyses are stored as member variables of the appropriate classes. A typical analysis workflow proceeds from loading the raw data, processing it, and performing a model fit. To enable this workflow, ModelFit has a member variable which is DataProcessed. Similarly, DataProcessed has a member variable which is DataRaw. This workflow design allows pyWitness to use information (parameters, computed statistics, etc.) from the previous processing step without user interaction. Otherwise, user interaction could potentially be a source of error. pyWitness object oriented design maximizes code reuse, which is critical for minimizing bugs and maximizing reproducibility. For example, signal detection-based model calculations only appear in one location in the code. ROC plots, simulated data generation, and optimization objective functions all use the same model code.

### Installing pyWitness

To install pyWitness, depending on the level of programming proficiency, users can either download the ZIP file, install via Conda (for less experienced users) or clone the GIT repository (for more advanced users). pyWitness uses standard packages from the python software ecosystem, including numpy (numerical arrays; Harris et al. (2020)), SciPy (fitting and functions; Virtanen et al. (2020)), pandas (data frames; McKinney (2010)), matplotlib (plotting; Hunter (2007a)), openpyxl (reading and writing excel; https://pypi.org/project/openpyxl/), xlrd reading and writing excel; https://xlrd.readthedocs.io/en/latest/ and numba (compiler to speed up code; Lam et al. (2015)). These dependencies are best installed using miniconda https://docs.conda.io/en/latest/miniconda.html. We tested the installation on Mac OS and Windows OS. The source code is stored in git (https://github.com/lmickes/pyWitness) and documented with Sphinx (https://www.sphinx-doc.org/en/master/).

### Manuscript structure

This paper has the form of a tutorial using example datasets. To show the features of pyWitness, we describe the steps of standard analyses. No single dataset can show the majority of the features of pyWitness. We therefore selected three datasets to demonstrate pyWitness, labeled test1, test2, and test3. The example datasets are also used in the online manual tutorials which expand on the descriptions given in this paper. The first dataset, test1, is of data collected on 6-person simultaneous lineups and has only one condition (Seale-Carlisle et al. , 2019a). The other two datasets each have two conditions. One dataset, test2, is data from 6-person simultaneous lineups, and the other dataset, test3, is data from showups (Experiments 1 and 3 from Wilson et al. (2018), respectively). These tutorial data are stored in the directory pyWitness/data/tutorial/, and users will need to navigate to this directory to follow the tutorial in this paper.

“Basic usage, and descriptive and inferential statistics” section explains how to load data, perform ROC analysis, CAC analysis, response time accuracy characteristic analysis (RAC), bin the raw data, and obtain descriptive statistics. “Model fitting” section explains how signal detection-based models are fitted to the processed data. “Advanced analysesand functionality” section briefly describes advanced functionality, including complex raw data loading and manipulating, bootstrapping raw data, and extending pyWitness. Once pyWitness has been installed, the code listings provided can be directly copied into a python terminal to produce the results presented in this paper. To avoid ambiguity and for completeness, code is often replicated in the listings.

## Basic usage, and descriptive and inferential statistics

Typically the first stage of an analysis is load experimental raw data and calculate some basic statistics to check the properties of the data sample. pyWitness allows users to separate data for different experimental conditions, to bin and pivot raw data. With the data binned and pivoted, they can then get correct ID (hit) and false ID (false alarm) rates, $d^{\prime }$ values, and partial area under the curve (pAUC) values. pyWitness also creates publication-ready ROC, CAC, and RAC plots.

### Data formats

To use pyWitness, the data must in a standard format. This can be achieved by using a data translator (a small fragment of Python code) or by transforming the raw data manually (e.g., in Excel or SPSS). Table 1 shows the mandatory data column names and allowed data types or values per column.

**Table 1:** Mandatory raw data columns and allowed values

lineupSize	targetLineup	responseType	confidence
integer	targetAbsent	suspectId	integer/float
	targetPresent	fillerId	
		rejectId	

Table 2 shows an example of the minimum data needed in a raw data file. Although there needs to be mandatory columns and variables, the file can include many more. For example, inclusion of conditions, demographic data, response time, etc. is acceptable. It is important to note that confidence has to be numerical (e.g., 1, 2 and 3) and not categorical (e.g., low, medium, and high) as with the latter there is no general way to order the data in ascending or descending confidence. Accumulating participant responses in decreasing confidence is needed for many of the commonly used eyewitness identification analyses. If the confidence collected was on a non-numerical scale, users will have to represent the categories as numbers.

**Table 2:** Example minimal raw data file

participant	lineup	target	response	confidence
Number	Size	Lineup	Type	
1	6	targetPresent	suspectId	6
2	6	targetAbsent	rejectId	9
3	6	targetPresent	rejectId	1
.	.	.	.	.
.	.	.	.	.

### Loading and checking raw data

pyWitness provides multiple ways for loading datasets. Listing 1 shows how a raw experimental data file (test1) in comma separated values (CSV) format is loaded as a pyWitness.DataRaw object called dr^1^
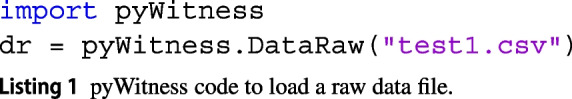


The first line imports the pyWitness package and the second line constructs a DataRaw object. Within the DataRaw constructor, the function dr.checkData() is called and displays the mandatory data columns and unique values in those columns. If additional columns are present, for example, test1 contains response time, the unique values can be displayed using dr.columnValues("responseTime"). Other files, such as Excel files, can also be loaded using dr = pyWitness.DataRaw("test1.xlsx").

### Processing raw data

For all analyses, the raw data need processing. The core processing consists of two steps. The first step is to create a frequency pivot table. In the table are rows of the different lineup types (target-absent and target-present) and participant responses (suspect ID, filler ID, and reject lineup). In the columns are confidence levels^2^. The second step is to cumulate the frequencies in decreasing confidence and compute correct and false ID rates rates. These processing steps are performed by calling the function dr.process() and it returns a pyWitness.DataProcessed object dp. To confirm that the processing steps worked and to see the values, the frequencies pivot and cumulative rates tables can be displayed by using dp.printPivot() and dp.printRates(), respectively. These commands are put together in Listing 2.
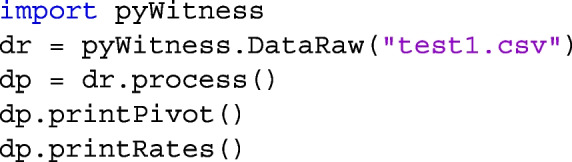


**Fig. 2:**
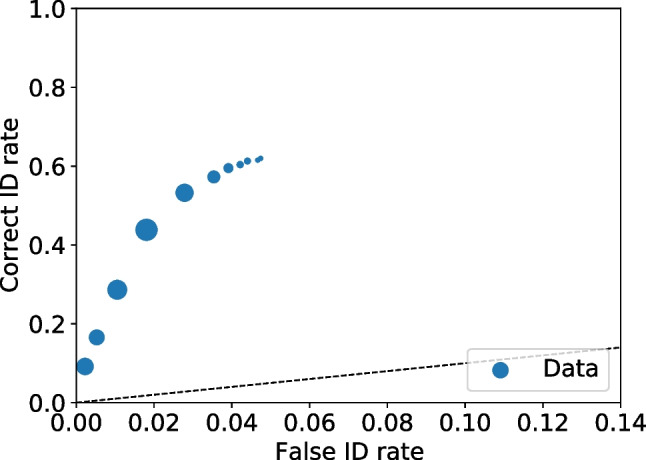
Test1 ROC. The point sizes reflect the relative frequency. The dashed line represents chance performance

With all the frequencies and rates available multiple values can be calculated, including relative frequencies (Seale-Carlisle et al. , 2019b), the correct ID and false ID rates, proportion correct, z-false alarms ($z_{FA}$) and z-hits ($z_{H}$), and $d^{\prime }.$ With the data processed, ROC and CAC curves can be plotted with dp.plotROC() and dp.plotCAC(), respectively. Figure 1 shows the ROC for the test1 data and Fig. 2 the corresponding CAC. All the plots in pyWitness are created using Matplotlib (Hunter , 2007b) and they can be formatted and tailored to the user’s needs.

**Fig. 3:**
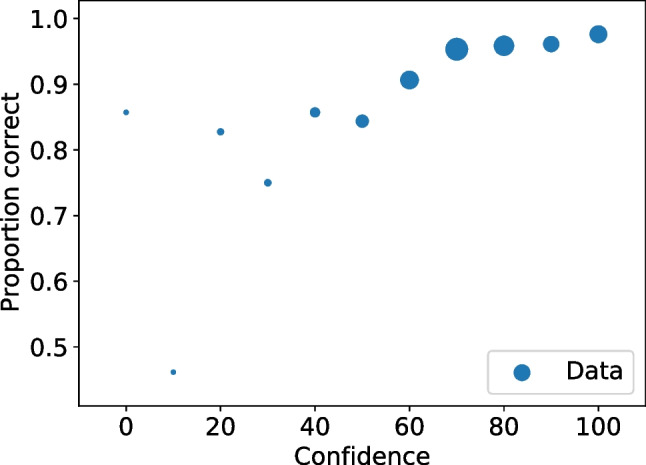
Test1 CAC. The point sizes reflect the relative frequency

pAUC is used to evaluate discrimination performance of an eyewitness (Mickes et al. , 2012; Gronlund et al. , 2014). pyWitness computes the pAUC by integrating (using Simpson’s rule) the correct and false ID rates up to an integration limit in the false ID rate (the default is to the maximum available false ID rate). The pAUC value is output by print(dp.pAUC). The user can specify the cutoff rate as a parameter for the process function. For example, if the pAUC needs to be calculated to a false ID cutoff of 0.04, then process needs to be called with dr.process(pAUCLiberal = 0.04). The two ways to collapse, or bin, data are by relabeling the categorical data or creating new labels by defining bins. In the test1 dataset, there are 10 confidence values, binning is performed on the raw data by calling the dr.collapseContinuousData function. An example of binning is given in Listing 3, where 3 bins are defined with confidence ranges (0, 60], (60, 80] and (80, 100] and labelled Low (1), Medium (2), and High (3), respectively.
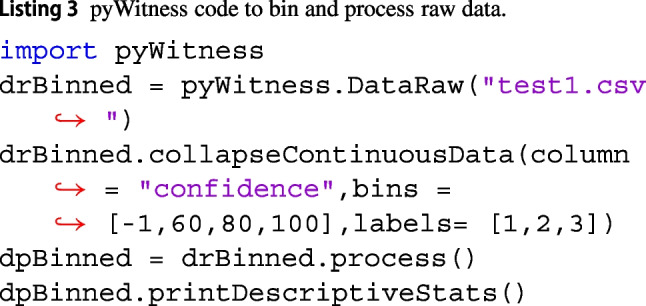


The frequencies of responses for each response type and descriptive statistics for the test1 dataset after binning are shown in Tables 3 and 4, respectively, produced by the functions printPivot() and printDescriptiveStats().

**Table 3:** Test1 frequencies of responses for each response type by level of binned confidence, produced by printPivot()

confidence		Low (1)	Medium (2)	High (3)
target-absent	filler ID	78	34	14
	reject ID	86	124	107
target-present	filler ID	21	15	6
	reject ID	49	42	37
	suspect ID	81	122	74

**Table 4:** Test1 descriptive statistics, produced by printDescriptiveStats()

	value
Number of lineups	890
Number of target-absent lineups	443
Number of target-present lineups	447
Correct ID rate	0.62
False ID rate	0.05
$d^{\prime }$	1.94
pAUC	0.02

**Fig. 4:**
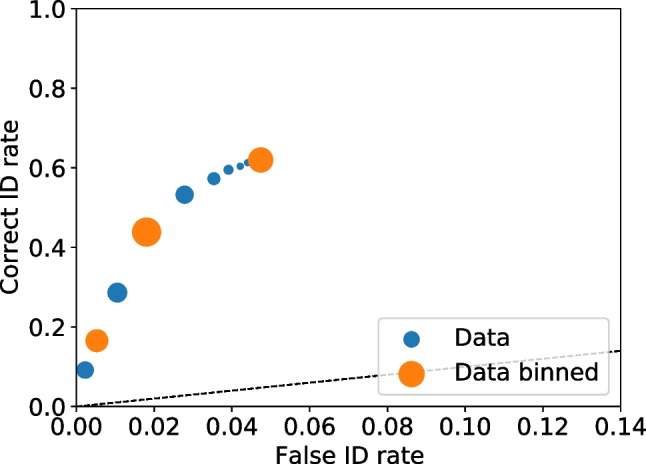
Test1 dataset unbinned and binned ROC. The point sizes reflect the relative frequency. The dashed line represents chance performance

Figures 3 and 4 show the binned and unbinned ROC and CAC plots for test1. After binning, the confidence on the horizontal axis of the CAC plot is the average reported confidence in each respective bin.

#### Response time analysis

**Fig. 5:**
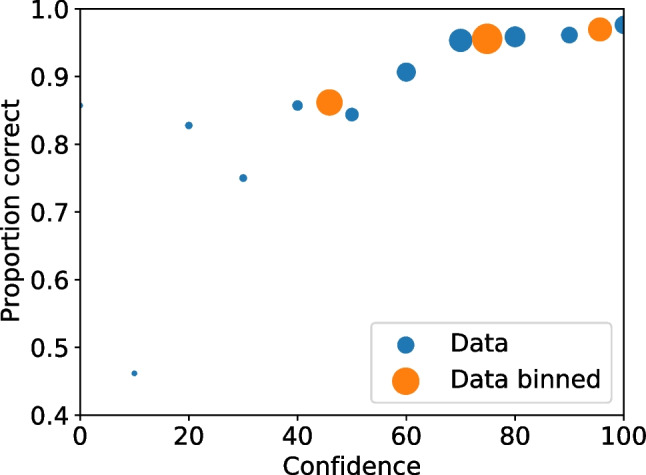
Test1 dataset unbinned and binned ROC. The point sizes reflect the relative frequency

pyWitness provides analyses of other dependent variables than confidence, including response time. It plots suspect ID accuracy by response time in RAC curves (Seale-Carlisle et al. 2019b). To perform this analysis (other dependent variables can be analysed and plotted this way), use the code in Listing 4. Response time analysis differs in the process function. When processing the raw data, the dependent variable needs to be defined, so the arguments dependentVariable =”responseTime” and reverseConfidence=True^3^ need to be provided to process. Figure 5 is the RAC plot with suspect ID accuracy (proportion correct) plotted against response time.
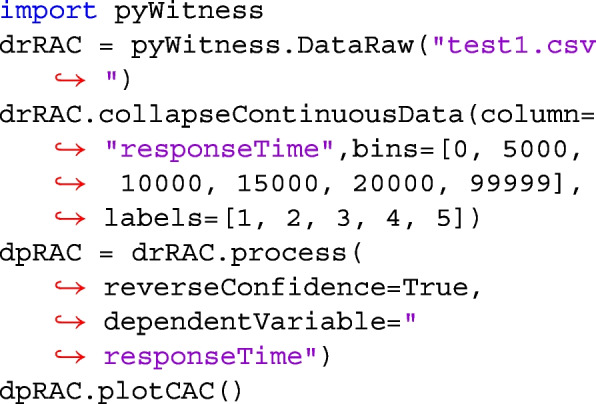


The example test1 dataset is of a single experiment with only one condition and no exclusions (e.g., participants who failed a validation test). With pyWitness, it is possible to select and process a raw data sub-sample. A sub-sample is selected using the dr.cutData(column,value) function and a particular experimental condition is processed by calling dr.process(column,value). How to use these methods are presented in examples in “Advanced analyses and function-ality” section on test2 and test3 datasets. These datasets have multiple conditions and data to be excluded.

**Fig. 6:**
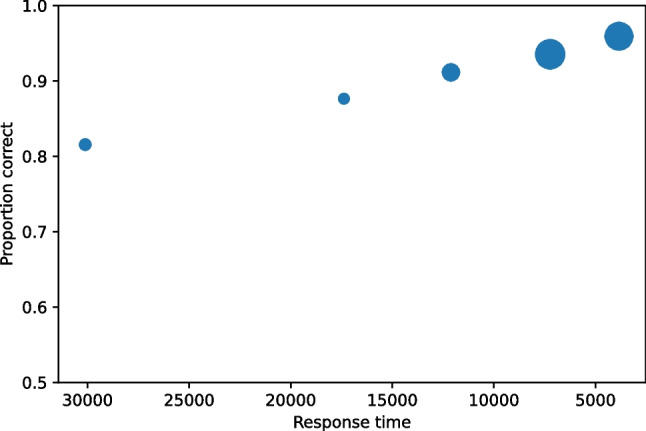
Test1 RAC. The point sizes reflect the relative frequency

## Model fitting

Different signal detection-based models have been adapted to fit lineup data (Wixted et al. , 2018). The models share general features, as shown in Fig. 6, of two Gaussian distributions (one for fillers and innocent suspects and the other for guilty suspects) along a memory strength axis. The lure distribution has mean $\mu _l$ and standard deviation $\sigma _l$ and the target distribution has mean $\mu _t$ and standard deviation $\sigma _t$. The figure shows an equal variance version of the model where $\sigma _t = \sigma _l$. In an unequal variance model, $\sigma _t$ does not have to equal $\sigma _l$.

**Fig. 7:**
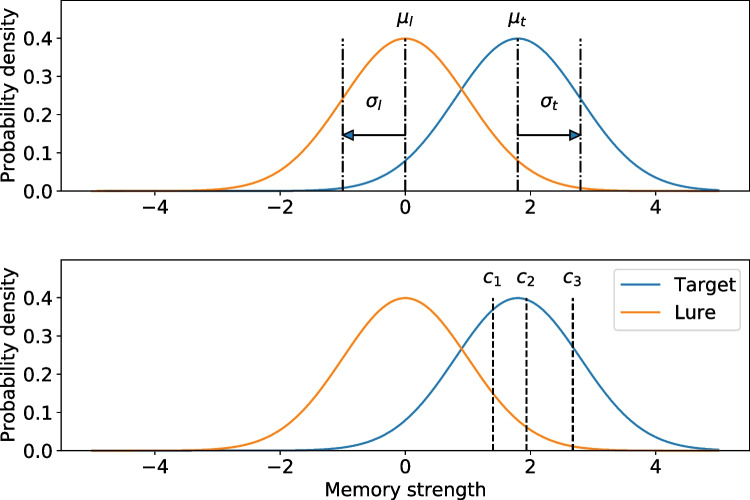
Equal variance signal detection model with a lure (innocent suspect and fillers) distribution with $\mu _l$ and $\sigma _l$ and target (guilty suspect) distribution with $\mu _t$ and $\sigma _t$ along a memory strength axis (top panel). The bottom panel shows the different criteria, $c_1$, $c_2$, and $c_3$ that represent most liberal criterion to most conservative criterion

In both the equal and unequal variance models, $c_1$, $c_2$, and $c_3$ represent different criterion that map onto confidence levels. If a memory signal strength exceeds $c_1$, but not $c_2$, the participant gives a low confidence response. If the memory signal strength exceeds $c_2$, but not $c_3$, the participant gives a medium confidence response. And if the memory signal strength exceeds $c_3$, the participant gives a high confidence response. If the memory signal strength does not exceed $c_1$, the participant makes no ID. To put it succinctly, when memory is tested on a lineup, if a memory strength signal is strong enough to exceed any criterion, the eyewitness will make an identification, otherwise they will reject the lineup.

The same applies when memory is tested on a showup or list-learning recognition memory test where if an item generates a signal strength that exceeds a criterion, then the participant gives an “old” (I saw the item on the study list), otherwise, a “new” (I did not see the item on the study list) response is made. The Gaussian distributions for a recognition memory test are of lures (similar to the filler and innocent suspect distribution) and targets (similar to the guilty suspect distribution).

Researchers can also designate an innocent suspect. To model the frequency of a selection of a designated innocent suspect a second lure distribution is required with mean $\mu _{l2}$ and standard deviation $\sigma _{l2}$. The decision rules are the same as in the two-distribution variant of the model. One can vary the designated innocent suspect distribution between the filler/lure distribution and the target distribution, this would enable modeling of unfair lineups. In simple terms, if $\mu _{l2}$ = $\mu _{t}$ and is greater than $\mu _{l}$, the innocent suspect resembles the guilty suspect more than the fillers (sign of an unfair lineup). If, $\mu _{l2}$ = $\mu _{l}$, the innocent suspect does not stand out among the fillers (sign of a fair lineup).

pyWitness implements fits for the simple independent observation model (for lineup, showup, and list-learning data), independent observation model, BEST-REST model, ensemble model and integration model (for lineup data). Table 5 shows 1) the definitions of the decision variables and rules, 2) the python commands to run the fit, and 3) whether memory strengths are correlated for each model. While the models have characteristics in common, the decision rules differ (Wixted & Mickes, 2018). Above, we described the MAX decision rule for the simple independent observation model.

**Table 5:** Models’ decision variables, decision rules, commands, and whether memory strengths are correlated

Model	Decision variable	Positive decision rule	Command	Correlated memory
				strength parameter
SIO	Memory strength	Identify the face that	ModelFitIndependentObservationSimple	No
	of a face	exceeds the decision criterion		
IO	Memory strength of a	Identify the MAX face if its memory	ModelFitIndependentObservation	Yes
	lineup member’s face	strength exceeds the decision criterion		
EN	Memory strength of a face	Identify the MAX face if its difference	ModelFitEnsemble	Yes
	minus the mean memory	score exceeds the decision criterion		
	strength of all the lineup			
	members’ faces			
BR	Memory strength of a face	Identify the MAX face if its difference	ModelFitBestRest	Yes
	minus the mean memory	score exceeds the decision criterion		
	strength of the other lineup			
	members’ faces			
IN	Memory strength values	Identify the MAX face if summed	ModelFitIntegration	Yes
	summed across all the	memory strength exceeds the		
	lineup members’ faces	decision criterion		

SIO is the simple independent observation model, IO is the independent observation model, EN is the ensemble model, BR is the best minus rest model, IN is the integration model

The other models also use the MAX decision rule. That is, the identified face is the one that yields the highest signal strength to exceed the criterion. For the Ensemble (Wixted & Mickes, 2014) and BEST-REST (Clark , 2003); Clark et al. (2011) models, the identified face is the one that yields a difference score of the face with the maximum memory strength and the average of memory strengths of all faces in the lineup that exceeds the criterion. For the integration model (Duncan , 2002), the identified face is the one that yields a sum of all memory strengths that exceed the criterion. For models with more complex decision rules than in the simple independent observation model, the criteria apply to the respective decision variable and not memory strength (e.g., in the bottom panel of Fig. 6, the decision making variable on the *x*-axis would be, for example, the sum of memory strengths for the integration model (Wixted et al. , 2018).

To implement the fits from these models, pyWitness has a generic framework that uses a single base class pyWitness.ModelFit. Specific models (e.g., independent observation model) are implemented as derived classes from the pyWitness.ModelFit superclass. The base class implements all of the parameter management, including calculating $\chi ^2$ values, fitting and minimizing, plotting functions, and generating data. The derived classes only need to implement the calculations for a particular model. For example, the concrete class for the independent observation model is ModelFitIndependentObservation. The derived fit classes only need to implement a function called calculateCumulativeFrequencyForCriterion that returns the predicted number of filler IDs and suspect IDs for target-absent and target-present lineups for each criterion value.

### Fit $\chi ^2$ calculation

pyWitness minimizes $\chi ^2$ calculated from comparing observed *O* and model predicted *E* values. pyWitness uses the standard SciPy minimizers. Model predictions are calculated for the number of target-absent lineups where a filler is identified (TAFID), target-absent lineups where the lineup is rejected (TARID), target-present lineups where a filler is identified (TPFID), target-present lineups where a suspect is identified (TPSID), and target-present lineups where the lineup is rejected (TPRID). The level of confidence is denoted by subscript *i*. For example, the expected number of lineup rejections in target-present lineups for a given level of confidence *i* is $E_{\textrm{TPRID},i}$. There are cases of lineups where there is a designated innocent suspect in target-absent lineups, if this suspect is identified it is a target-absent suspect ID (TASID). The innocent suspect identifications are usually estimated for fair lineups from the target absent filler IDs. The three $\chi ^2$ calculations for different types of data are described below.

#### Lineups without a designated innocent suspect

For data without a designated innocent suspect, the $\chi ^2$ used by the minimizer is the sum of the following contributions

1 $$\begin{aligned} \chi ^2_\text {TAFID}= & {} \sum _{i=1}^{N} \frac{\left( O_{\text {TAFID},i} - E_{\text {TAFID},i} \right) ^2}{E_{\text {TAFID},i}}, \end{aligned}$$

2 $$\begin{aligned} \chi ^2_\text {TPFID}= & {} \sum _{i=1}^{N} \frac{\left( O_{\text {TPFID},i} - E_{\text {TPFID},i} \right) ^2}{E_{\text {TPFID},i}}, \end{aligned}$$

3 $$\begin{aligned} \chi ^2_\text {TPSID}= & {} \sum _{i=1}^{N} \frac{\left( O_{\text {TPSID},i} - E_{\text {TPSID},i} \right) ^2}{E_{\text {TPSID},i}}, \end{aligned}$$

4 $$\begin{aligned} \chi ^2_\text {TARID}= & {} \frac{\left( O_{\text {TARID}} - E_{\text {TARID}} \right) ^2}{E_{\text {TARID}}}, \end{aligned}$$

5 $$\begin{aligned} \chi ^2_\text {TPRID}= & {} \frac{\left( O_{\text {TPRID}} - E_{\text {TPRID}} \right) ^2}{E_{\text {TPRID}}}, \end{aligned}$$

where *N* is the number of confidence bins, yielding a total $\chi ^2$ for a lineup $\chi ^2_{\text {lineup}}$ of

6 $$\begin{aligned} \chi ^2_{\text {lineup}}= & {} \chi ^2_{\text {TAFID}} + \chi ^2_{\text {TPFID}} + \chi ^2_{\text {TPSID}} \nonumber \\{} & {} + \chi ^2_{\text {TARID}} + \chi ^2_{\text {TPRID}}. \end{aligned}$$

The number of degrees of freedom ${\text {ndf}}_{\text {lineups}}$ is given by $3N-N_ {\text {parameters}}$. $N_{\text {parameters}}$ is the number of fit parameters.

#### Lineups with a designated innocent suspect

For data with a designated innocent suspect, an additional innocent suspect lure distribution is considered by the additional contribution of $\chi ^2_\textrm{TASID}$

7 $$\begin{aligned} \chi ^2_{\text {TASID}} = \frac{\left( O_{\text {TASID},i} - E_{\text {TASID},i} \right) ^2}{E_{\text {TASID},i}}, \end{aligned}$$

making the total $\chi ^2_{\text {unfair-lineup}}$ to

8 $$\begin{aligned} \chi ^2_{\text {unfair-lineup}}= & {} \chi ^2_{\text {TAFID}} + \chi ^2_{\text {TPFID}} + \chi ^2_{\text {TPSID}} \nonumber \\{} & {} + \chi ^2_{\text {TARID}} + \chi ^2_{\text {TPRID}} + \chi ^2_{\text {TASID}}. \end{aligned}$$

The number of degrees of freedom, $\text {ndf}_{\text {unfair-lineups}}$, is given by $4N-N_{\text {parameters}}$.

#### List-learning and showups

For data from list-learning recognition memory tests (i.e., participants study a list of target items and test on the targets and lures items) and showups (where participants are shown the guilty suspect, target-present, or the innocent suspect, target-absent) there are only two contributions

9 $$\begin{aligned} \chi ^2_\textrm{TA}= & {} \sum _{i=1}^{2N} \frac{\left( O_{\textrm{TA},i} - E_{\textrm{TA},i} \right) ^2}{E_{\textrm{TA},i}}, \end{aligned}$$

10 $$\begin{aligned} \chi ^2_\textrm{TP}= & {} \sum _{i=1}^{2N} \frac{\left( O_{\textrm{TP},i} - E_{\textrm{TP},i} \right) ^2}{E_{\textrm{TP},i}}, \end{aligned}$$

to the total $\chi ^2_\textrm{showup}$,

11 $$\begin{aligned} \chi ^2_\textrm{showup} = \chi ^2_\textrm{TA} + \chi ^2_\textrm{TP}. \end{aligned}$$

For target-present or target-absent trials where the participant rejects with confidence $c_i$, the confidence level is inverted to a negative value, hence, the sum ranges from 1 to 2*N*. The number of degrees of freedom $\textrm{ndf}_\textrm{showup}$ is given by $4N-N_\textrm{parameters}$.

### Calculating expected values

For the different forms of data, each model must produce the expected number of participant responses, so the *E* values. The following sections describe how the expected values are calculated in pyWitness.

#### Lineups without a designated innocent suspect

The current state-of-the-art models for lineup data are relatively complex, so we consider a simple signal detection-based model for a lineup with *n* lineup members for illustrative purposes. Performing a fit involves calculating the cumulative likelihoods *L* ($L_\textrm{TAFID}$, $L_\textrm{TPFID}$, and $L_\textrm{TPSID}$) for different models. Calculating each of the likelihood values is fundamentally an integral over memory strength of a signal detection-based model. The decision rule for this simple model is that there is a single memory strength for a target that is larger than any lure memory strengths. The cumulative (i.e., summing or integrating up to a confidence level or criterion) likelihoods $L_i$ for the simple model are

12 $$\begin{aligned} L_{\textrm{TAFID},i}= & {} n \int _{c_i}^{\infty } \varPhi (x;\mu _l,\sigma _l)^{n-1} \phi (x;\mu _l,\sigma _l) \; dx, \end{aligned}$$

13 $$\begin{aligned} L_{\textrm{TPFID},i}= & {} (n-1) \int _{c_i}^{\infty } \varPhi (x;\mu _l,\sigma _l)^{n-2} \varPhi (x;\mu _t,\sigma _t)\nonumber \\{} & {} \phi (x;\mu _l,\sigma _l) \; dx, \end{aligned}$$

14 $$\begin{aligned} L_{\textrm{TPSID},i}= & {} \int _{c_i}^{\infty } \varPhi (x;\mu _l,\sigma _l)^{n-1} \phi (x;\mu _t,\sigma _t) \; dx, \end{aligned}$$

where $\phi$ is a Gaussian probability density function and $\varPhi$ is a Gaussian cumulative density function.

Equation 12 is the likelihood of filler IDs for target-absent lineups. The integrand is *n* independent probabilities drawn from the lure distribution. Equation 13 is the likelihood of filler IDs for target-present lineups. The integrand is $n-2$ draws from the lure distribution, multiplied by a draw from the target distribution and a draw from the lure distribution. Equation 14 is the likelihood of suspect IDs for target-present lineups. The integrand is $n-1$ draws from the lure distribution and a single draw from the target distribution.

The structure of the integrals for lineup models with a more complex decision rule (independent observation, BEST-REST, ensemble, or integration models) are similar to those of the simple model. The main differences are the integration interval and a model dependent term *F* (Wixted et al. , 2018) that encodes the decision rule. The likelihoods are given by

15 $$\begin{aligned} L_{\textrm{TAFID},i}= & {} \int _{-\infty }^{\infty } \varPhi (x;\mu _l,\sigma _l)^{n-1} \phi (x;\mu _l;\sigma _l) \nonumber \\{} & {} F(x;c_i,\mu _l,\sigma _l,n) \; dx,\end{aligned}$$

16 $$\begin{aligned} L_{\textrm{TPFID},i}= & {} (n-1) \int _{-\infty }^{\infty } \varPhi (x;\mu _l,\sigma _l)^{n-2} \varPhi (x;\mu _t,\sigma _t)\nonumber \\{} & {} \phi (x;\mu _l,\mu _l) F(x;c_i,\mu _l,\sigma _l,n) \; dx,\end{aligned}$$

17 $$\begin{aligned} L_{\textrm{TPSID},i}= & {} \int _{-\infty }^{\infty } \varPhi (x;\mu _l,\sigma _l)^{n-1} \phi (x;\mu _l;\sigma _l) \nonumber \\{} & {} F(x;c_i,\mu _l,\sigma _l,\mu _t,\sigma _tt,n) \; dx. \end{aligned}$$

In general, the calculation of *F* is rather involved and thus not described in this paper, pyWitness implements the appropriate terms from Wixted et al. (2018).

The expected number in a given confidence bin can be calculated from two likelihoods between two criteria and multiplying by the number of lineups,

18 $$\begin{aligned} E_{\textrm{TAFID},i}= & {} N_\textrm{TA} \left( L_{\textrm{TAFID},i} - L_{\textrm{TAFID},i-1} \right) ,\end{aligned}$$

19 $$\begin{aligned} E_{\textrm{TPFID},i}= & {} N_\textrm{TP} \left( L_{\textrm{TPFID},i} - L_{\textrm{TPFID},i-1} \right) ,\end{aligned}$$

20 $$\begin{aligned} E_{\textrm{TPSID},i}= & {} N_\textrm{TP} \left( L_{\textrm{TPSID},i} - L_{\textrm{TPSID},i-1} \right) , \end{aligned}$$

where $N_{TA}$ is the number of target-absent lineups and $N_{TP}$ is the number of target-present lineups.

The expected number of lineup rejections is calculated by summing the all of the IDs and taking the difference between the number of target-present $N_\textrm{TP}$ and target-absent $N_\textrm{TA}$ lineups,

21 $$\begin{aligned} E_\textrm{TARID}= & {} N_{TA} - E_\textrm{TAFID} - E_\textrm{TASID},\end{aligned}$$

22 $$\begin{aligned} E_\textrm{TPRID}= & {} N_{TP} - E_\textrm{TPFID} - E_\textrm{TPSID}, \end{aligned}$$

where *E* values without subscripts are the sum over all confidence levels, for example

23 $$\begin{aligned} E_\textrm{TAFID} = \sum _i E_{\textrm{TAFID},i}. \end{aligned}$$

#### Lineups with a designated innocent suspect

For data from lineups with a designated suspect in target-absent lineups, extra expected values $E_{\textrm{TASID},i}$ are required. The simple version of the likelihood (which would correspond to Eqs. 12, 13 and  14) for this case is given by

24 $$\begin{aligned} L_{\textrm{TASID},i} = \int _{c_i}^{\infty } \Phi (x;\mu _l,\sigma _l)^{n-1} \phi (x;\mu _{l2},\sigma _{l2}) \; dx, \end{aligned}$$

where $\mu _{l2}$ and $\sigma _{l2}$ are the mean and standard deviation of the third Gaussian distribution for the innocent suspects in target-absent lineups. In fair lineups with a designated innocent suspect, there is no statistical value in separately fitting the TASID.

#### List-learning and showups

For list-learning and showup data the simple independent observation model is applicable. The model is simplified when $n=1$, so $L_{\textrm{TPFID},i}=0$ and $L_{\textrm{TAFID},i} = L_{\textrm{TASID},i}$, so Eqs. 12, 13 and 14 reduce to

25 $$\begin{aligned} L_{\textrm{TASID},i}= & {} \int _{c_i}^{\infty } \phi (x;\mu _l,\sigma _l) \; dx ,\end{aligned}$$

26 $$\begin{aligned} L_{\textrm{TPSID},i}= & {} \int _{c_i}^{\infty } \phi (x;\mu _t,\sigma _t) \; dx . \end{aligned}$$

This model is the standard signal detection model described in classic texts or papers (e.g., Macmillan & Creelman, 2005).

### Fit example

pyWitness greatly simplifies the process of fitting models to data. Code Listing 5 shows an example of an independent observation model fit in pyWitness. The model fit mf is constructed with a pyWitness.DataProcessed object, called dp in the example.
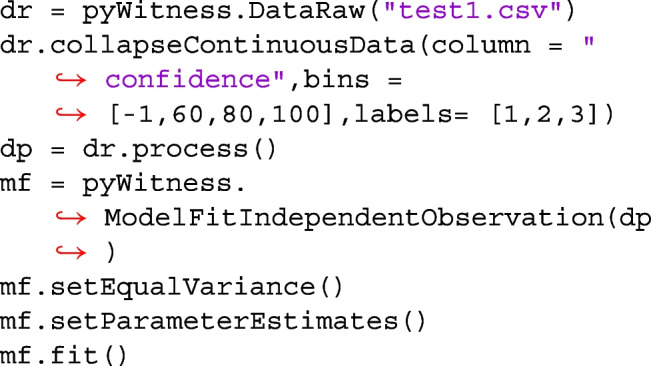


**Fig. 8:**
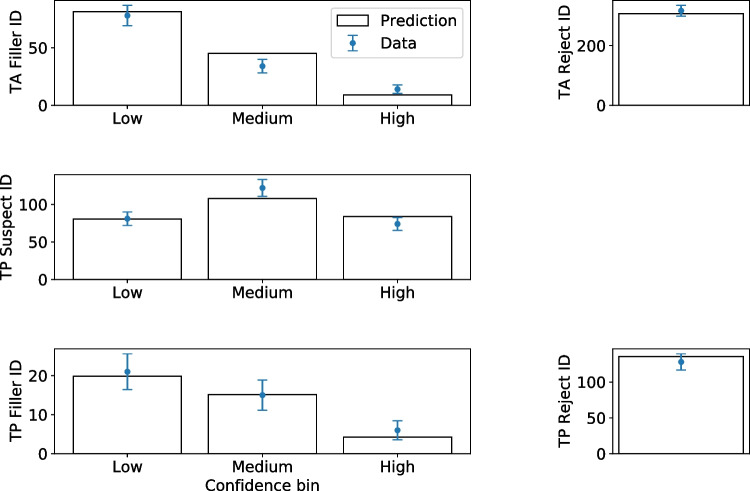
Histograms of observed and fitted expected frequencies using the equal variance independent observation model for test1 data

**Fig. 9:**
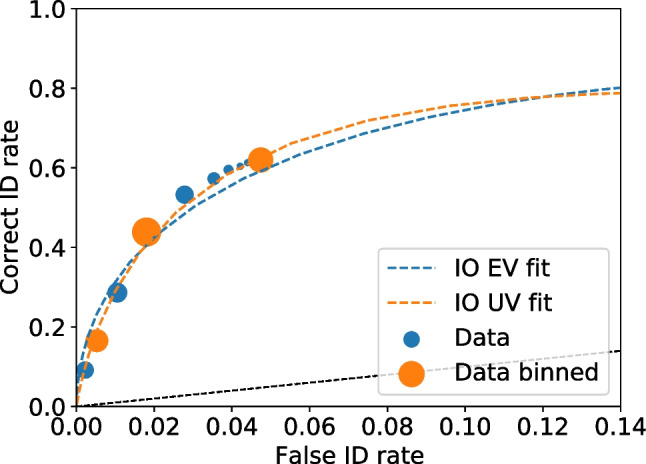
ROC for unbinned and binned test1 data. The dashed lines are the equal (EV) and unequal (UV) independent observation (IO) models fits on the binned data. The point sizes reflect the relative frequency. The black dashed line represents chance performance

Figure 7 shows the comparison between the observed and expected frequencies for test1 data for each response type and confidence bin. The plot is created after a fit has finished by calling mf.plotFit(). Similarly the signal detection-based model, like the one shown in Fig. 6, can be created by calling mf.plotModel(). Figures 8 and 9 show the ROC and CAC from the model fit compared with data.

Table 6 shows the fit parameters and the information about the fitting (e.g., number of iterations and fit time) for the available models, including equal and unequal variance models for test1 data. All models fit the data adequately. The number of iterations ranged from 89 to 435 and the fit times range from 7.39 to 48.07 seconds. Performing this number of fits is trivially easy and fast with pyWitness, the results are highly reproducible, and requires minimal user input.

### Controlling fits

A key feature of pyWitness is the ability to control model fitting using an easy to use python code interface which aids reproducibility. Controlling the convergence of signal detection-based models can be difficult due to the large number of fit parameters. In addition to controlling the fit convergence, modellers require the ability to allow parameters to vary, set to fixed values, or set to be equal to each other. pyWitness has a simple code interface to the control the behavior of the fit. The python class ModelFit stores all the fit parameters as Parameter objects. A Parameter is the aggregation of the parameter name string Parameter.name, a numerical value Parameter.value, a Boolean flag to indicate if the parameter is free to vary or fixed Parameter.fixed, and a link to another parameter object Parameter.other.

Using a fit object to store the model parameters and functions to execute the fit, has many advantages over a function. One advantage is that the fit can be stopped, parameter or parameters adjusted, and the fit restarted. Another advantage is that multiple ModelFit objects can be used concurrently, for example one with equal variance and another with unequal variance. This design also makes it simple to perform a simultaneous fit to mulitple data sets using the same parameters.

In the model fit code in Listing 5, the ModelFit has a method setEqualVariance. A simplified version of the method (only the lure and target Gaussian parameters) is given in Listing 6. The model has a lure distribution mean (lureMean) and sigma (lureSigma) and target mean (targetMean) and sigma (targetSigma). The lureMean is fixed and set to value of 0, the lureSigma is set equal to the targetSigma. The targetMean is free to vary and is set to initial value of 1, and the targetSigma is fixed to a value of 1.
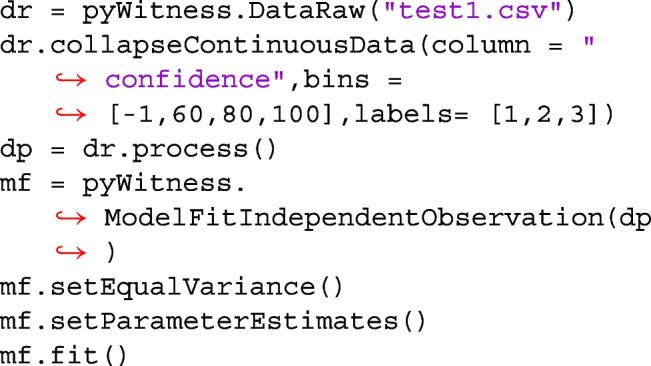

**Fig. 10:**
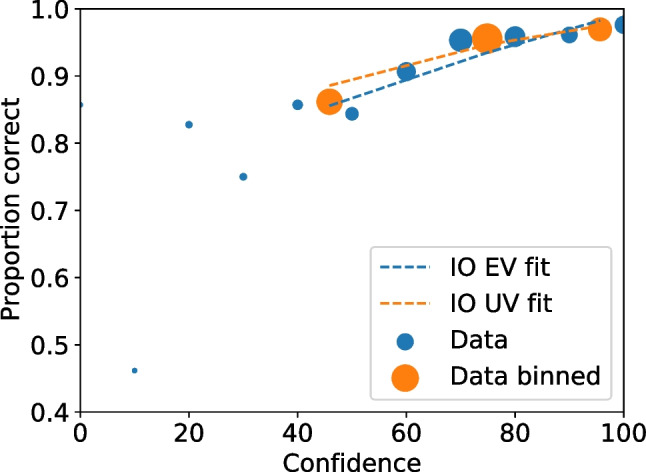
CAC for unbinned and binned test1 data. The point sizes reflect the relative frequency. The dashed lines are the equal (EV) and unequal (UV) independent observation model (IO) fits on the binned data

Using these Parameters, a user can create their own functions or input code directly into the python command line to constrain fits according their requirements.

Fits with a large number of criteria tend to take a long time to converge. This convergence can be substantially accelerated by a suitable estimation of the fit starting parameters. pyWitness does this by computing a *z*-ROC of the processed data. The *z*-ROC ($z_H$ and $z_{FA}$) and signal detection ($\sigma _t$ and $\mu _t$) parameters are related by

27 $$\begin{aligned} z_H = \frac{z_{FA} + \mu _t}{\sigma _t}, \end{aligned}$$

where $z_H$ is the *z*-score of the correct ID rate and $z_{FA}$ is the *z*-score of the false ID rate. Starting parameters for the fits can be determined by fitting a linear function to $z_H$ against $z_{FA}$, the *z*-ROC. The gradient is the target sigma and the intercept with the vertical axis is the target mean. The fit starting parameters can be set from the *z*-ROC by calling ModelFit.setParameterEstimates().

**Table 6:** Signal detection-based model fit parameters and number of iterations and fit time for dataset test1

Parameter	SIO-EV	SIO-UV	IO-EV	IO-UV	BR-EV	BR-UV	EN-EV	EN-UV	IN-EV	IN-UV
$\mu _l$	0	0	0	0	0	0	0	0	0	0
$\sigma _l$	1.00	1.00	1.00	1.00	1.00	1.00	1.00	1.00	1.00	1.00
$\mu _t$	1.95	1.96	1.80	1.89	2.04	2.04	2.04	2.04	2.54	2.73
$\sigma _t$	1.00	0.74	1.00	0.79	1.00	0.61	1.00	0.61	1.00	1.44
$\sigma _{b}$	-	-	0.60	0.45	-	-	-	-	-	-
$c_1$	1.62	1.65	1.40	1.54	1.74	1.75	1.45	1.46	1.23	1.28
$c_2$	2.10	2.08	1.94	2.00	2.21	2.17	1.84	1.81	2.77	2.90
$c_3$	2.80	2.67	2.68	2.63	2.95	2.76	2.46	2.30	4.79	5.07
ndf	4	3	4	3	4	3	4	3	4	3
$\chi ^2$	20.15	8.79	10.30	4.53	23.20	7.87	23.20	7.87	12.78	7.35
$\chi ^2/\textrm{ndf}$	5.04	2.93	2.58	1.51	5.80	2.62	5.80	2.62	3.20	2.45
*p*-value	0.001	0.032	0.036	0.209	0.000	0.049	0.000	0.049	0.012	0.062
number of iterations	89	141	189	353	100	164	105	182	435	435
fit time [s]	7.39	15.15	7.47	14.55	12.18	17.74	12.57	19.41	15.10	48.07

SIO = simple independent observations model, IO = independent observations model, BR = best-rest model, EN = ensemble model, IN = integration model, EV = equal variance, UV = unequal variance

The method ModelFit.fit() calls a standard scipy.optimize routine. The method has the signature ModelFit.fit(maxiter,method,resetParameters). The argument maxiter specifies the maximum number of iterations (the default is 5000), method is the name of the minimization algorithm passed onto scipy.optimise (the default is ‘Nelder-Meade’), and resetParameters in a optional flag to reset the parameters to their default values.

The ModelFit class gives the user the convenient ability to store the fit history. For example, the parameters and $\chi ^2$ values for each step in the minimization is stored to diagnose non-convergent fits or to investigate false minima. Once a fit has converged, the fit parameters with their associated uncertainties can be displayed by using the ModelFit.printParameters() method.

The fitting has been tested on a wide range of publicly available eyewitness ID data. These datasets can be found in the pyWitness code repository. Furthermore, we have performed comparison tests between the models in pyWitness with models in R and MATLAB (Wixted et al. , 2018) and achieved excellent agreement.

## Advanced analyses and functionality

“Basic usage, and descriptive and inferential statistics” and “Model fitting” sections provided a walk-through of a complete analysis using data from a single experiment with a single condition (test1). This section describes more advanced features of pyWitness, e.g., bootstrapping and pAUC statistical comparisons. To walk-through these features requires more complex data, including data that start with a more complex data format and has two conditions per experiment. In this advanced tutorial, we use data from Wilson et al. (2018), referred to as test2 and test3.

### Converting raw data formats and data translators

There is large degree of diversity in the format that experimental data is stored. To perform analysis of these data samples, there needs to be a clearly documented and reproducible translation or reformatting process. The data format used by pyWitness is based on the format used by sdtlu Cohen et al. (2021). If translation of the data is simply a one-to-one mapping of column names and their values stored in each column pyWitness.DataRaw has methods to perform the mapping. Otherwise a data translator (a small fragment of python code) is needed, therefore a wide range of translators are provided in the class pyWitness.DataTranslator.

### Including or excluding data

DataRaw has a method to include or exclude data from subsequent analyses, called DataRaw.cutData. In Listing 7, the data in which the column labelled "previouslyViewedVideo" is equal to 1 is kept within the DataRaw object.
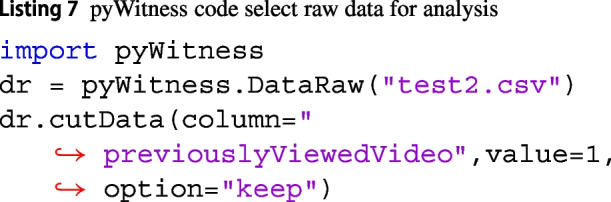


The option can be either "keep" or "cut". The DataRaw.cutData can be called multiple times to obtain the data sample required for subsequent analyses.

### Extracting data for different experimental conditions

Processing raw data for different experimental conditions is similar to including (option="keep") or excluding data (option="cut"). Below in Listing 8 is an example of selecting data in a “control” condition and “verbal” condition.
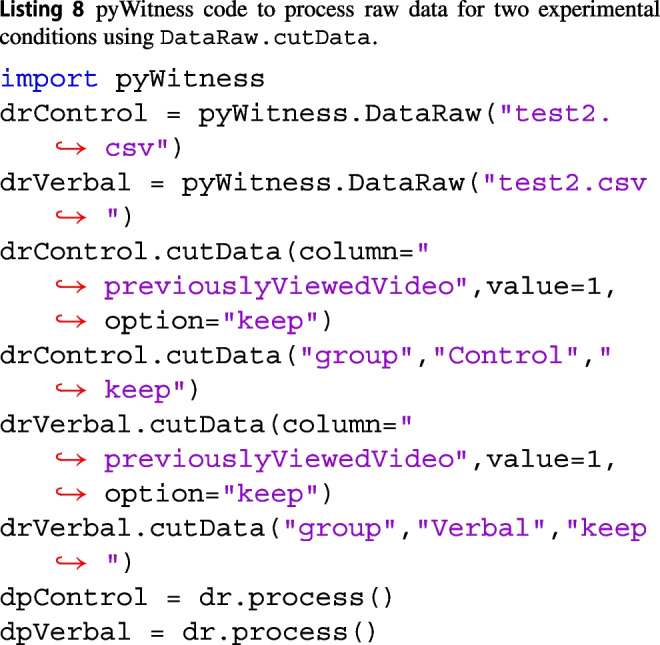


The DataRaw.process method can also be used to select data for processing. In Listing 9, the column and value arguments for the DataRaw.process method are used to select a subset of the raw data. This is equivalent to the DataRaw.cutData described above.
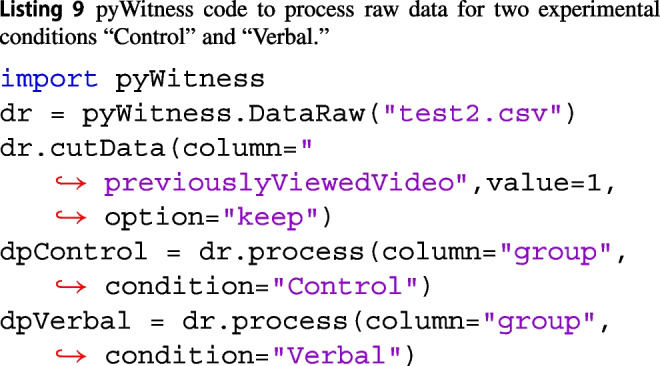


dpControl and dpVerbal are instances of DataProcessed and can be used for all analyses described in this paper.

### Computing bootstrapped confidence intervals

pyWitness uses the bootstrap method to determine uncertainties (e.g., confidence intervals) on descriptive and model-dependent statistics. Replica data are created from the original data with replacement. These replicas are processed using the full pyWitness analysis pipeline, as the entire analysis can be automated. Listing 10 shows how to compute 95% confidence intervals using 2000 bootstrap replicas.
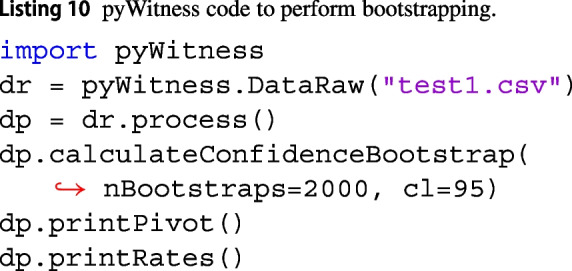


The distributions of the final quantities are used to calculate confidence intervals. Table 7 shows the 95% confidence limits of test1 descriptive statistics computed using 2000 bootstrap replicas.

**Table 7:** Test1 descriptive statistics with bootstrapped 95% confidence intervals

Statistic	Value	Lower 95% CL	Upper 95% CL
Correct ID rate	0.619	0.568	0.663
False ID rate	0.047	0.039	0.054
$d^{\prime }$	1.941	1.765	2.107
pAUC	0.021	0.016	0.025

**Fig. 11:**
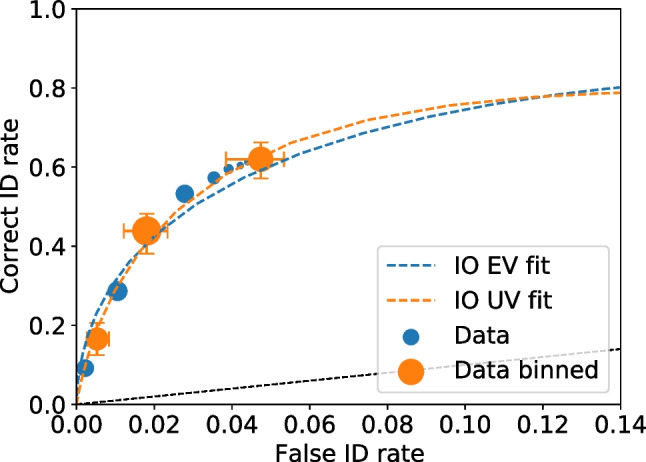
Test1 ROC for unbinned and binned data with uncertainties on the binned data. The point sizes reflect the relative frequency. The dashed lines are the equal and unequal independent observation model (IO) fits on the binned data. The black dashed line represents chance performance

Given all values are recomputed for each bootstrap replica, it is straightforward to compute confidence intervals on cumulative rates, such as those needed for ROC and CAC plots. Figures 10 and 11 show the ROC and CAC plots, respectively, with the 95% confidence limits indicated as error bars.

**Fig. 12:**
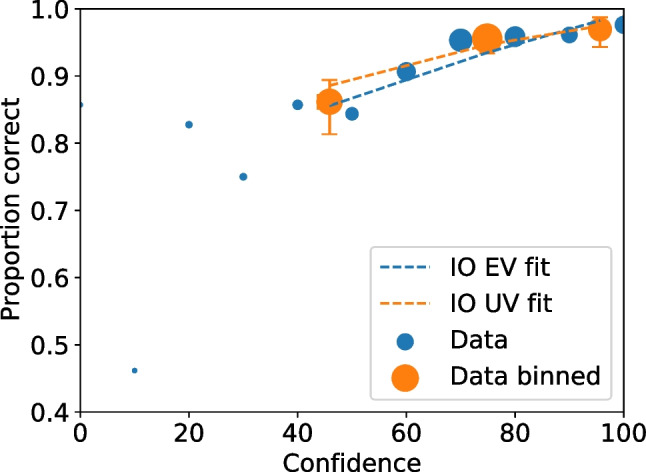
Test1 CAC for unbinned and binned data with uncertainties on the binned data. The point sizes reflect the relative frequency. The dashed lines are the equal (EV) and unequal (UV) independent observation model (IO) fits on the binned data

### Statistical partial area under the curve tests

To statistically compare two ROC curves, the partial area under the curve (pAUC) for each group or condition is computed and then compared. The approach taken in pyWitness is the same as in pROC R-package (Turck et al. , 2011), a *Z* score is computed as

28 $$\begin{aligned} Z = \frac{\textrm{pAUC}_1 - \textrm{pAUC}_2}{sd(\textrm{pAUC}_1 - \textrm{pAUC}_2)}, \end{aligned}$$

where $\textrm{pAUC}_1$ and $\textrm{pAUC}_2$ are the pAUCs from the two conditions to be compared. The standard deviation in the denominator is computed from all the bootstrap replicas. The *Z* score is then used for a one-tailed *Z*-test.

The test2 example dataset consists of data from two groups, called “control” and “verbal.” The overall false ID rate will generally not be the same for two groups. The data for both groups have to be processed first to find the most conservative condition (the cutoff often used). Listing 11 shows a two pass processes, the first processing is to find the two cutoffs and then the second pass is to compute pAUC with a consistent cutoff.
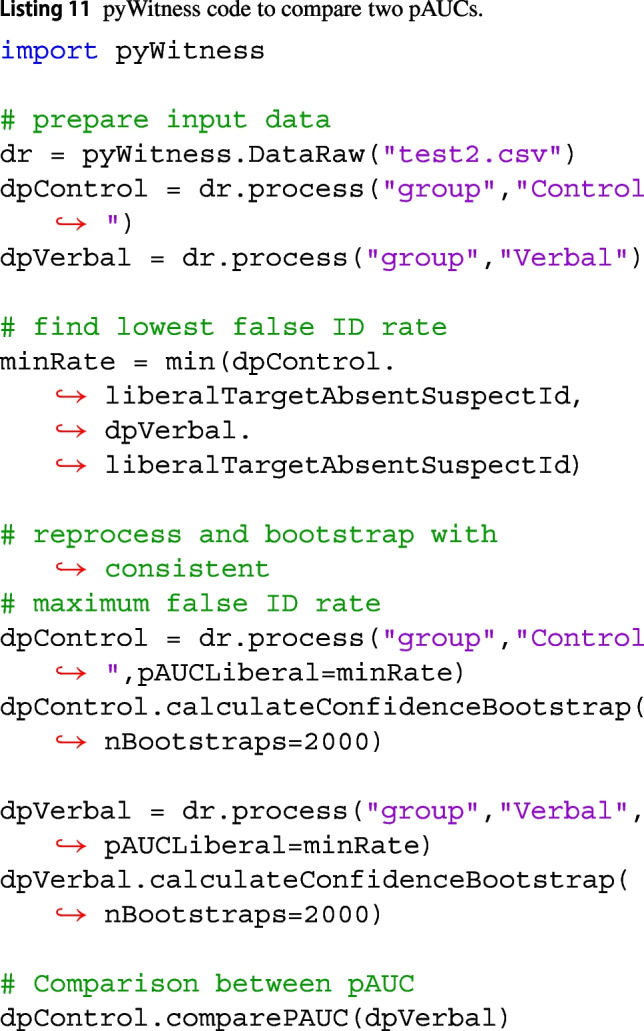


For test2 data the smallest, most conservative overall false ID rate is 0.09 (i.e., the rightmost point of the verbal ROC curve). The output of dpControl.comparePAUC, gives the pAUC for the control group of 0.0259 with a standard error of 0.004 and pAUC for the verbal group of 0.0310 with a standard error of 0.003. Using Eq. 28 yields a *Z* value of 1.16 with a p-value of 0.247. This is consistent with results reported in Wilson et al. (2018).

**Fig. 13:**
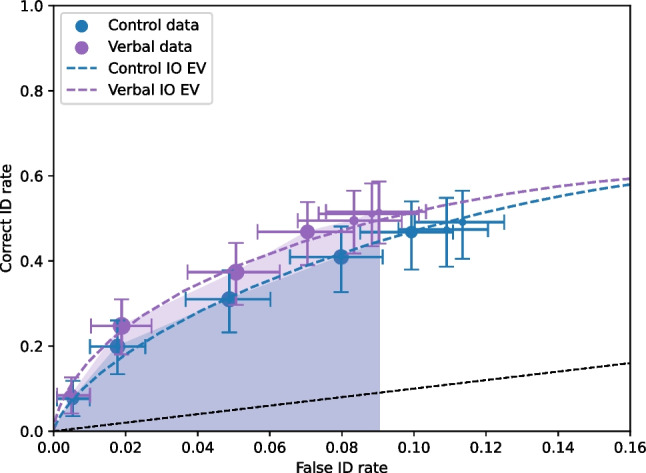
Test2 ROC curves for two conditions. The shaded regions show the pAUCs that were compared; in this example, the false ID rate of the condition that yielded the most overall conservative responding was used as the cut-off point. The dashed colored curves represent the equal variance independent observations model (IO EV) fits per condition. The error bars are 95% confidence intervals. The point sizes reflect the relative frequency. The dashed line represents chance performance

The ROC curves can be plotted to show the pAUCs used for the comparison. Figure 12 shows the ROC curves of data of the two conditions (test2 dataset). Much information is provided in this plot. The compared pAUCs are shown by the shaded regions, including the false ID cut-off rate. The verbal condition yielded the most conservative responding overall. Therefore, the cut-off for measuring pAUC was the overall false ID rate from that condition (0.09). The relative frequencies are reflected by point sizes, so the smaller points show fewer responses than the larger points. Uncertainties are 95% confidence levels. Equal variance independent observation model fits for each condition are also presented.

### Showup analyses

We use the test3 dataset as an example of a showup experiment. A showup is a one-person ID procedure where only the innocent or guilty suspect is presented. This procedure is much like a standard list-learning recognition memory experiment with old (targets) and new (lures) items. In the case of the showup, the innocent suspect is new, and the guilty suspect is old. However, unlike in a recognition memory experiment, where participants are tested on tens to hundreds of trials, participants are typically only tested on one showup trial. The processing for showup data is exactly the same as for lineup data (see Listing 2), the input data has a mandatory column, lineupSize, which is set to 1 for showup data. This notifies pyWitness that the data are from showups.

Figure 13 shows the ROC curve of the test3 showup dataset. With showup and list-learning data, the entire range of the ROC is plotted, not truncated as with lineup data (i.e., there is no lineup rejection associated with a particular face in a lineup, e.g., “they are not in the lineup,” thus only old responses are plotted).

**Fig. 14:**
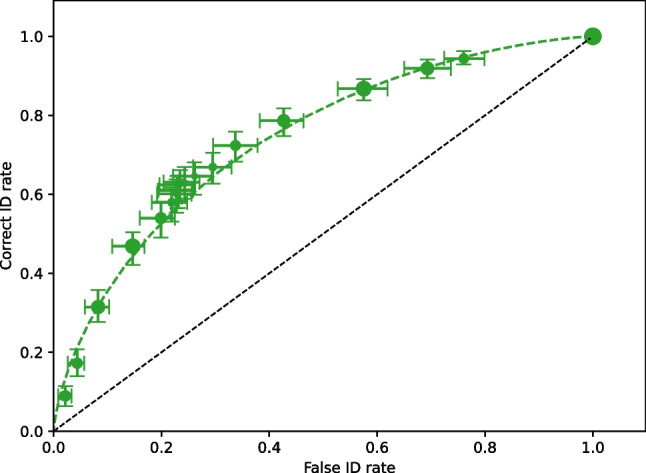
Test3 ROC curve with 95% confidence intervals. The black dashed line represents chance performance. The dashed curve is the equal (EV) independent observation model (IO) fit. The point sizes reflect the relative frequency

### Simulating data and power analyses

It is possible to simulate raw data from either processed data or models. Both DataProcessed and ModelFit have a method called generateRawData(nGenParticipants), which creates a new set of simulated data with nGenParticipants. The number of responses in each category (rejectID, fillerID, suspectID) are generated from a multinomial distribution. Using the basic fit example in Listing 5, Listing 12 is expanded to generate simulated data from the fit model.
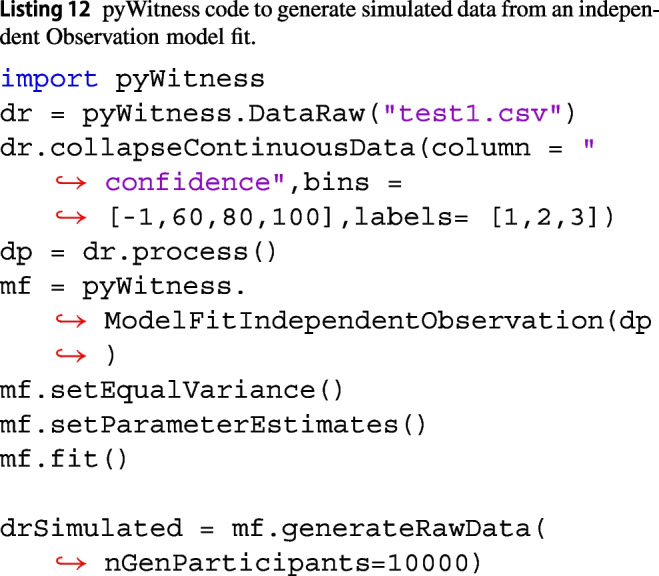


The drSimulated object is an instance of DataRaw, which allows all analyses to be performed. Examples where this feature is critical are model recovery fits and power analyses.

Listing 13 is a simple power analysis where simulated data are generated for 500, 1000, 1500, 2000, ... 5000 participants and the pAUC with its confidence limits is computed.
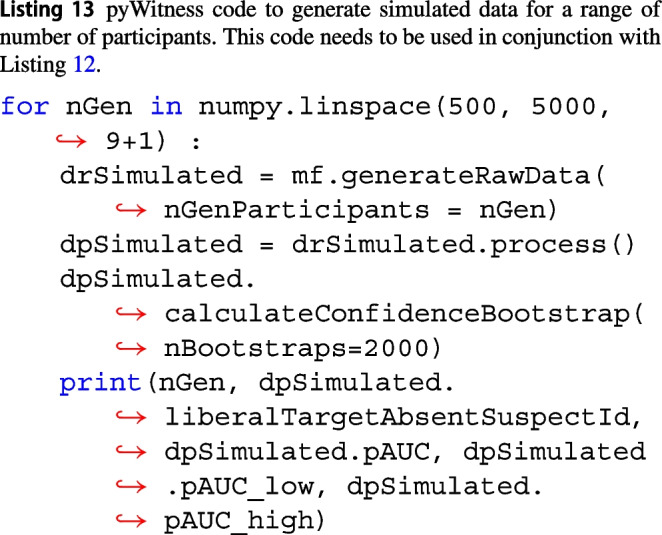


Currently, designated innocent suspect lineups cannot be simulated, but will be added in a future release.

### Extending pyWitness

pyWitness is designed to be extended and adapted. Two extensions that would be particularly useful for users are complex raw data import and manipulation, and new models.

Users may want to import data that is not easily translated to pyWitness format, if, for example, the data need to be manipulated in a non-trivial manner or there is complex logic needed to exclude participants. The DataRaw class has a member variable DataRaw.data that is of type pandas.DataFrame. Users familiar with pandas can create their own pandas.DataFrame and assign it to the member variable of DataRaw.data or directly manipulate DataRaw.data.

A new signal detection inspired model can be created quickly in pyWitness. Each model is a Python class derived from ModelFit and must implement at least two methods, a constructor and ModelFit.calculateCumulativeFrequencyForCriterion(self,c). The argument c is a floating point number for the criterion and the function returns a list of $E_\textrm{TAFID}$, $E_\textrm{TPFID}$ and $E_\textrm{TPSID}$.

## Future directions

pyWitness allows for many potential innovations in analysis and modelling of eyewitness ID and list-learning data. This paper describes the current version 1.0 of the code. Many extensions are possible, including confidence intervals on fit parameters, full Monte Carlo simulations of data with complex decision rules, binned and unbinned maximum likelihood fits, Bayesian analyses, and advanced power analyses. sdtlu also implements a parameter for base rates^4^ (the proportion of lineups that contain a guilty suspect) and sequential lineups with a stopping rule(s), these features can be easily added to pyWitness, and will be in version 2.0. Beyond eyewitness ID research, the code can also already be applied to general memory (as measured with standard list-learning recognition memory tasks) and visual perception (e.g., detection-plus-localization visual search tasks; Wixted et al. (2021)). This section briefly describes the planned future developments of pyWitness.

Estimating the uncertainties on the fit parameters remains a challenge for signal detection-based modelling of eyewitness identification data. The processes for bootstrapping and subsequently fitting the replicas is straightforward in pyWitness, but still thousands of fits are required. Each individual fit can be optimized using techniques described in this paper, for example using the *z*-ROC estimates for the target and lure distribution parameters and other heuristics for estimating the criteria thresholds before fitting (Selker et al. , 2019). Assuming a time per fit of 10 s would result in total computation time for 2000 bootstraps of 5.6 hours. Given each replica is independent from another, computational time can be reduced using simple parallel processing methods, for example dividing the problem over multiple processors on modern multi-core CPUs.

The integrations performed for all of the signal detection-based model fits for lineups have assumptions in order to simplify the integration over memory strength. This approach is computationally efficient but might affect the final results. A Monte Carlo approach, where many lineups are fully simulated assigning probabilities from each distribution (lure, target, and lure2, for lineups with a designated suspect) and a decision rule formulated on the basis of the *n* memory strengths might be the simplest way to construct models without assumptions. pyWitness provides a framework to carry out the Monte Carlo simulations and will be included in a future release. The main issue with this approach is the computational speed when performing fits. Again modern parallel programming practices can come to the rescue.

With the current $\chi ^2$ minimization function used in pyWitness, the expected frequency for a given confidence bin cannot be zero. It might be possible to use the observed variance, and pyWitness does allow this via an optional parameter to the fitting functions, but still the observed variance potentially could also be zero. It is the users’ responsibility to appropriately bin the data so that the $\chi ^2$ is well-defined. This requirement can be avoided by using maximum likelihood as opposed to $\chi ^2$. This also has the added benefit of not making an assumption of Gaussian uncertainties when there are low numbers of participant responses in a given bin.

“Simulating data and power analyses” section outlines how a power analysis can be performed using pyWitness. As implemented in the current version, the user needs to understand and use most of the functionality of pyWitness to perform a power analysis. A useful development is a simplified and streamlined power analysis function that can quickly estimate the power required for future studies based on existing verified models. We are investigating deploying a dashboard application based on pyWitness.

## Concluding remarks

pyWitness is a powerful tool to process experimental eyewitness identification and recognition memory data, perform current state-of-the-art model fits, perform standard ROC statistical tests, and simulate experimental data. pyWitness gathers together in a single package many of the most current eyewitness signal detection-based models and serves as a reference implementation for future eyewitness memory research. The code was designed to be simple enough for an early career researcher without any modelling experience to use quickly, yet flexible enough for an expert to extend. The output of pyWitness is numerical (e.g., pAUC statistical tests), excel spreadsheets (e.g., pivot table frequencies and rates), and publication-ready plots.

We developed pyWitness with replication and reproduction in mind. The code has been extensively tested on publicly available experimental data. The code since inception has been stored in git and freely available from github (https://github.com/lmickes/pyWitness). The online manual (https://lmickes.github.io/pyWitness/) is a mixture of restructured text and automatic documentation generated from the comments within the python code. The code is distributed with a set of regression tests that allow a user or developer to compare the code against previously obtained results.

As described in the “Future directions” section there are many developments which could extend pyWitness. It is our hope that a community of researchers and modellers will use and extend pyWitness and future developments are likely to be community driven. Many of the future plans exist in development form, but are not yet validated and ready for use. These developments will appear in the code repository and online manual as soon as possible.

We believe that reproducible and easily communicated analyses, open science practices, training of early career researchers in modelling and programming should not be a chore, but an integral part of psychological science practice and we demonstrate this as much as possible with pyWitness.

## Footnotes

1 The variables used for objects in code listings are typically an abbreviation, so a DataRaw object is stored as dr. A user can choose any variable name they prefer.

2 We use “confidence,” but it does not have to be confidence, it could be another variable, such as response time.

3 Reverse confidence is set to true as the response time is usually smaller for more confident responses.

4 Most experiments use a base rate of 0.5 base, so half of the lineups contain the guilty suspect.
